# The Subchronic Toxic Effects of *Mosla chinensis Maxim* in Normal Rats

**DOI:** 10.1155/2020/4521586

**Published:** 2020-12-28

**Authors:** Dan Lei, Longxue Li, Shenghong Huang, Li Liu, Pingdong Cai, Zhouyang Gu, Kun Shu, Shouming Li, Tao Hong, Zhiyong Liu

**Affiliations:** ^1^Jiangxi University of Chinese Medicine, Nanchang 330004, China; ^2^Jiangxi Medical Service Guidance Center, Nanchang 330006, China; ^3^School of Science and Technology, Jiangxi University of Traditional Chinese Medicine, Nanchang 330004, China; ^4^Jiangxi Children's Hospital, Nanchang 330006, China; ^5^Jiangxi Provincial Key Laboratory of Traditional Chinese Medicine Pharmacology, Nanchang 330004, China

## Abstract

**Background:**

The aim of this work was to study the toxic effects and target organs of Mosla chinensis Maxim (MCM) in rats and provide theoretical basis for clinical medication.

**Methods:**

The subchronic toxicity study was conducted on 60 male and female SD rats using the fixed-dose method for the treatment groups and 20 male and female SD rats for the control. At the subchronic toxicity study, the water extract of MCM with fixed doses of 0.2 g/kg/day, 2 g/kg/day, and 20 g/kg/day was administered for 90 days intragastric, and the control group was given the same amount of distilled water. After 90 days, the general conditions of the rats were observed. Assessment on safety of the extract was conducted by a subchronic toxicity test which mainly examined alteration occurrence in gut flora and urine metabolism.

**Results:**

There was no significant difference in physical signs, reactivity, and stool characteristics in the four groups. Compared with the control group, the number of red blood cells in the male 2 g/kg/day group and the female 0.2 g/kg/day group was significantly different (*P* < 0.05). The detection of serum biochemical indicators showed that MCM has an effect on liver and kidney function but has no physiological significance. The level of low-density lipoprotein in male rats was lower than that in the control group (*P* < 0.05). Compared with the control group, the blood glucose levels of female rats in the 0.2 g/kg/day, 2 g/kg/day, and 20 g/kg/day groups were significantly increased (*P* < 0.05). As far as the diversity of intestinal flora is concerned, feeding MCM for 90 days has an influence on the distribution of intestinal flora. The content of lactic acid bacteria increased, and the ratio of hard bacteria to Bacteroides (f/b) was also affected, but there was no significant difference.

**Conclusions:**

These findings showed that the long-term intragastric administration of the MCM is safe to use within its dose recommendation. But it could have a slight effect on the metabolism of uric acid by changing the composition of intestinal flora and affecting the metabolism of tryptophan.

## 1. Background

Mosla chinensis Maxim cv. Jiangxiangru (MCM) is mainly distributed in Jiangxi province. Also, as the unique resource in Jiangxi, it belongs to the member of the Labiatae family and is usually used for both food and medicine. The whole herb can be used as medicine and is clinically used to treat acute gastroenteritis, abdominal pain, and diarrhea. The main chemical components of Elsholtzia are volatile oil, followed by flavonoids and glycosides, triterpenes and steroids, and a small amount of coumarins, lignans, cyanogenic glycoside, organic acids, and aliphatic hydrocarbons. Its volatile oil has many pharmacological effects, such as pesticides, antioxidants, and antibacterial [1]. It is one of the components of Changyanning, a medicine for gastroenteritis [2]. Due to its significant anti-inflammatory and other active effects, MCM has been listed as a traditional Chinese medicine with both medicinal and food functions. However, when some Chinese herbal medicines are transformed and metabolized by the liver, themselves or their metabolites can directly or indirectly cause liver damage or cause toxic damage to hepatocytes through immune-mediated mechanisms [3]. For example, Chinese herbal medicines such as Polygonum multiflorum, Celandine, Xiaochaihu decoction, and Huangyaozi have potential liver toxicity [4]. For scientific evaluation of the safety of MCM and mastering its security, in this study, the water extract of MCM was used. Through 90 days of gastric administration, the changes of intestinal flora were observed by using 16S DNA sequencing technology, and the changes of urine metabolism were observed by UHPLC-MS, in order to provide the basis for comprehensively understanding the safety evaluation of MCM.

## 2. Materials and Methods

### 2.1. Experimental Animals

The SD rats originated from the National Laboratory Animal Seed Center and bred at the Experimental Animal Science and Technology Center of Jiangxi University of Traditional Chinese Medicine, of which half were male and half were female, 8 weeks old, and weighed 100 ± 10 g. Experimental rats were raised in a SPF barrier system. In this experiment, a barrier housing facility was used in accordance with the national standard laboratory animal requirements of environment and housing facilities. The rats were housed in acrylic cages lined with wood shavings at constant room temperature (23 ± 1°C) and maintained on a 12 h light and 12 h dark cycle. The care of the laboratory animals and the animal experimental operation were performed in accordance with the committee of the Jiangxi University of Traditional Chinese Medicine.

### 2.2. MCM Water Extract Extraction

The experimental drug was purchased from Jiangzhong Traditional Chinese Medicine Decoction Pieces Co., Ltd. It was identified as the aerial part of the labiform plant Elsholtzia MCM, and the decoction was prepared by the pharmacy department of the affiliated hospital, and the liquid equivalent of the dry herb is 1.5 : 1.

### 2.3. Experimental Apparatus

The following instruments were used: automatic blood coagulation analyzer (Japan Sysmex Corporation, version: Japan Sysmex, CA-1500), automatic biochemical analyzer (American Beckman Coulter, version: Beckman Coulter AU480), inverted microscope (Japan Nikon, version: TS100-F), electronic balance (Ihaus/Shanghai version: CP114), multifunctional microplate reader (Thermofidel Technology Co., Ltd. Version: Varioskan Flash), high-speed refrigerated centrifuge (Shanghai Sai Murphysal Technology Co., Ltd.), UltiMate 3000 ultra-high performance liquid chromatography system, and LTQ Orbitrap Velos Pro high-resolution mass spectrometer instrument (Thermo Fisher Company).

### 2.4. Experimental Reagents

The following were used: ALT (alanine transaminase), AST (aspartate aminotransferase), ALP (alkaline phosphatase), GGT (glutamyl transferase), TB (total bilirubin), DB (direct bilirubin), TP (total protein), ALB (albumin), CHE (cholinesterase), TBA (total bile acid), UA (uric acid), TG (triglyceride), TC (total cholesterol), HDL (high-density lipoprotein), LDL (low-density lipoprotein), GLU (glucose), ADA (adenosine deaminase), CRE (creatinine), BUN (blood urea nitrogen) level detection kit (Shanghai Kehua Biotechnology Co., Ltd.), MDA (malondialdehyde), LPO (lipid peroxide), TNF (tumor necrosis factor), IL-1 (interleukin1), GAS (gastrin), Mtl (motilin), Na+-K+-ATP, CGRP (calcitonin gene-related peptide), ET-1 (endothelin1) level of blood detection kit (provided by Shanghai Hepai Biotechnology Co., Ltd.). HPLC grade methanol and acetonitrile were purchased from Anaqua (Wilmington, DE USA). Distilled water was obtained from a Milli-Q Ultrapure water system (Millipore, Billerica, MA, USA).

### 2.5. Animal Exposure

Eighty SD rats, half male and half female, were randomly divided into eight groups, consisting of a normal control group (C), 0.2 g/kg/day group (L), 2 g/kg/day group (M), and 20 g/kg/day group (H). Thus, each group consisted of 20 rats. All rats were acclimatized for one week and given normal food and distilled water. The treatment was conducted according to the following procedures. In the normal control group, animals were administered normal food and equal amount of distilled water without MCM water extract. Water MCM extract at doses of 0.2, 2, and 20 g/kg/day was given to the L, M, and H groups, respectively. The entire administration period was 90 days. It was administered per os once a day (1 ml/100 g b.w.). The body weight of the rats was recorded once a week, and the dose of MCM was adjusted according to the new body weight. At the beginning of the experiment, the difference in animal weight was not over or under 10% of the average weight.

### 2.6. Observation Indicators

#### 2.6.1. Observation of Clinical Manifestations

We observe the clinical manifestations of rats in the cage after gavage every day, including health status, feeding and drinking behaviors, food intake, and the color of the hair and fur.

#### 2.6.2. Biochemistry Analysis

At the end of the experiment, blood routine and liver and kidney biochemical function tests were done. Rats were anesthetized with 2% pentobarbital sodium, and blood samples were collected from the femoral artery for hematology and blood biochemical examination.

#### 2.6.3. Determination of Immunology, Gastrointestinal Motility, Antioxidation, and Energy Metabolic Capacity

The levels of rat serum immunoglobulin (IgA, IgM, and IgG) and complement (C3 and C4) were measured by detection kits. The intestinal kinetics, oxidation, and energy metabolism indexes of Na^+^-K^+^-ATP, MDA, LPO, TNF, IL-1, GAS, Mtl, CGRP, and ET-1 were measured by ELISA assay.

#### 2.6.4. Histological Examination

Rats were dissected after blood collection, and organs including the heart, liver, spleen, lung, kidney, stomach, thymus, pancreas, adrenal gland, brain, male testis epididymis, female ovary, and uterus were collected and weighed. Then, they were fixed and stored with 4% formaldehyde solution. All organs were washed and dehydrated, paraffin embedded, sectioned, stained with hematoxylin-eosin (HE), and observed under an optical microscope, and routine pathological examinations were performed.

#### 2.6.5. Determination of Intestinal Flora

Rats were fasted on the day before dissection, and feces were collected from the anus by tail-lifting stimulation, placed in a cryotube, and frozen at -80°C, and the sequence examination of intestinal flora was conducted in the Shanghai Majorbio Bio-pharm Technology Co., Ltd.

#### 2.6.6. Component Identification of Urine

UHPLC analysis was performed on an Ultimate 3000 UHPLC system (Thermo Scientific, San Jose CA, USA), and the chromatographic separation was carried out using ACQUITY UPLC BEH C18 1.7 *μ*m column (2.1 × 100 mm, Waters Corporation, Ireland). The UHPLC mobile phase consisted of 0.1% aqueous formic acid (solvent A) and acetonitrile (solvent B). The gradient duration is presented in Table S1. The column temperature was kept constant at 40°C. A blank sample is applied between every ten samples to wash the column.

An orbitrap mass spectrometer (LTQ Orbitrap Velos Pro, Thermo Fisher Scientific, San Jose, CA, USA) equipped with a heated electrospray ionization (HESI) probe was coupled to the UHPLC system. Detection was performed in both positive and negative ionization. The resolution type was set to 60000 FWHM, and all samples were operated on under the full scan mode. Dynamic exclusion was set to exclude a precursor ion for repeated MS/MS analysis within 15 s. The activation type was collision-induced dissociation (CID), and the intensity threshold was set at 1000. Other ion-source parameters of MS experiments were set as follows: heater temp, 350°C; sheath gas flow rate, 35 arb; aux gas flow rate, 10 arb; sweep gas flow rate, 0 arb; I spray voltage, 3.6 kV; capillary temp, 320°C; mass range, m/z 100-1000.

### 2.7. Statistical Analysis

Data were tested for significance between groups using IBM SPSS 26.0 application software, and single-factor ANOVA was used to test body weight, blood routine, blood biochemical indicators, and ELISA data. The UPLC-MS data were analyzed using SIMCA (Umetrics Company, USA), and also, they were analyzed using principal component analysis (PCA) and partial least squares discriminant analysis (PLS-DA) for multivariate statistical analysis. PCA, an unsupervised multivariate statistical approach, can reduce the dimensionalities of complex datasets and provide an overview of all observations in data tables. PLS-DA is used to reveal the net treatment effect on the subjects to detect the ions that have the greatest effect on the variance. Potential biomarkers were explored on the basis of variable importance in the project (VIP > 1) value. VIP value of PLS-DA plays a major role in separation of the factors. The one-way analysis of variance (ANOVA) was applied to measure the significance of each metabolite. Metabolites with both multivariate statistical significance (VIP > 1) and univariate statistical significance (*P* < 0.05) were considered to be markers responsible for the differentiation of the MCM treatment group from the control group. Finally, the ion spectrum of potential biomarkers was matched with the structure message of metabolites acquired from the Human Metabolome Database HMDB. Also, please refer to the following databases: METLIN (http://metlin.scripps.edu/), KEGG (http://www.kegg.com/), and mzCloud (https://www.mzcloud.org/). GraphPad Prism 8.0.2 application software was for graphing software.

## 3. Results

### 3.1. Effects of MCM Administration on Clinical Manifestations

The results showed that there were no significant physical toxicity signs and symptoms in behavior, activity, hair and fur color, food intake, drinking water, external reaction, and feces between the experimental group and the normal control group.

### 3.2. Effects of MCM Administration on Body Weight

**Figure 1:**
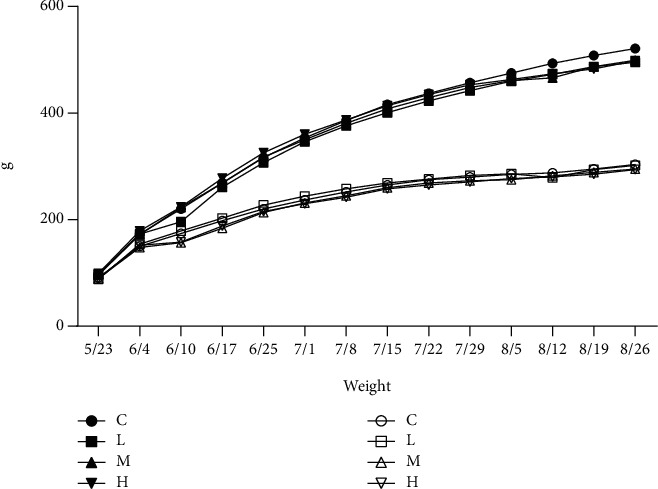
Effect of MCM on body weight in rats (*n* = 10). The hollow shapes represent female rat groups and solid shapes represent male rat groups. C: normal control group; L: water extraction of MCM 0.2 g/kg/day; M: water extraction of MCM 2 g/kg/day; H: water extraction of MCM 20 g/kg/day.

As shown in Figure 1, the body weight of all groups increased with the feeding time, and there was no obvious difference between the experimental groups and the normal control group (*P* > 0.05).

### 3.3. Effects of MCM Administration on Routine Blood Analysis

**Figure 2:**
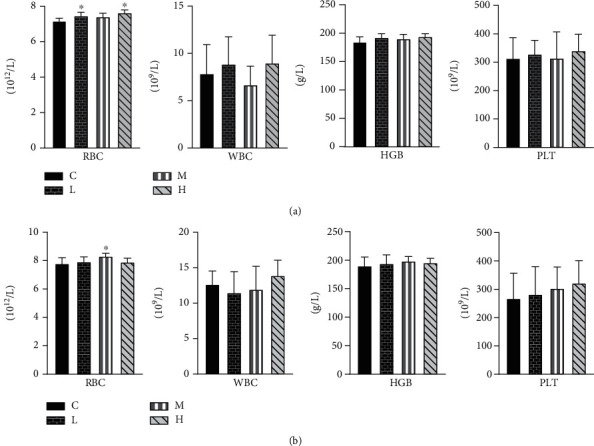
Effect of MCM on routine blood in rats. (a) represents female rat groups and (b) represents male rat groups. C: normal control group; L: water extraction of MCM 0.2 g/kg/day; M: water extraction of MCM 2 g/kg/day; H: water extraction of MCM 20 g/kg/day.c ^∗^*P* < 0.05, compared with the control group; ^∗∗^*P* < 0.01, compared with the control group. MCM: Mosla chinensis Maxim; RBC: red blood cell; WBC: white blood cell; HGB: hemoglobin; PLT: platelet.

The blood routine test results of rats, after 90 days of administration, are shown in Figure 2. The numbers of red blood cells in the 0.2 and 20 g/kg/day female groups significantly increased compared with the female control group (*P* < 0.05) (Figure 2(a)), and the male rats in the 2 g/kg/day group were significantly increased compared with those in the male control group (Figure 2(b)) (*P* < 0.05). No significant abnormalities were found in the number of other cells.

### 3.4. Effect of MCM on Serum Biochemical Index

**Figure 3:**
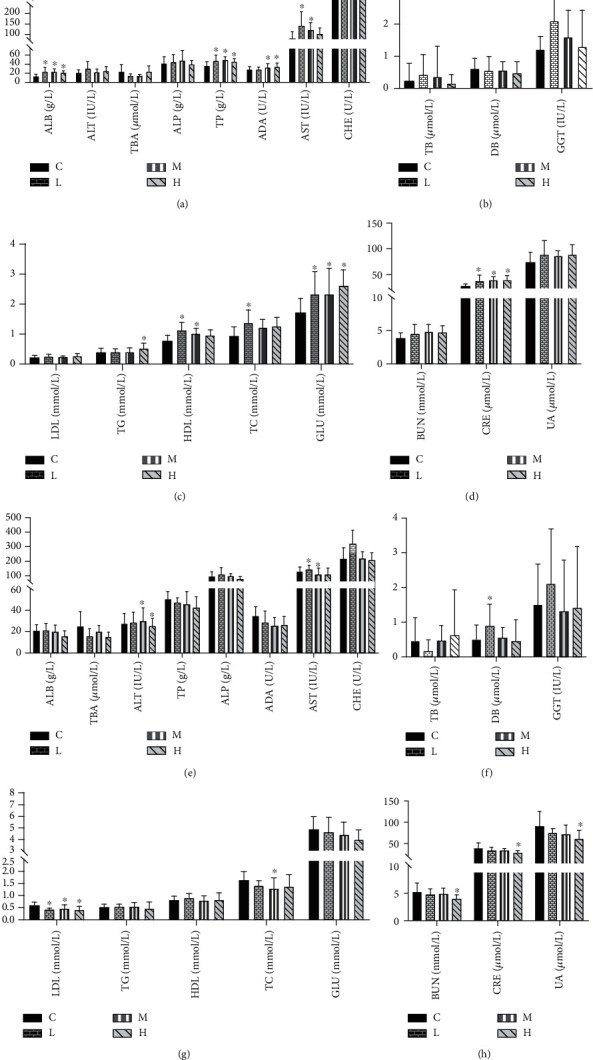
Effect of MCM on liver and kidney function-related indexes, blood glucose, and blood lipids in rats. (a–d) represent female rat groups and (e–h) represent male rat groups. C: normal control group; L: water extraction of MCM 0.2 g/kg/day; M: water extraction of MCM 2 g/kg/day; H: water extraction of MCM 20 g/kg/day. ^∗^*P* < 0.05, compared with the control group; ^∗∗^*P* < 0.01, compared with the control group. ALB: albumin; TBA: total bile acid; ALT: alanine transaminase; TP: total protein; ALP: alkaline phosphatase; ADA: adenosine deaminase; AST: aspartate aminotransferase; CHE: cholinesterase; TB: total bilirubin; DB: direct bilirubin; GGT: glutamyl transferase; LDL: low-density lipoprotein; TG: triglyceride; TC: total cholesterol; HDL: high-density lipoprotein; GLU: glucose; BUN: blood urea nitrogen; CRE: creatinine; UA: uric acid.

After the end of administration, serum was collected from each rat for liver and kidney biochemical index detection, and the results are shown in Figure 3. The levels of ALT, GGT, ALP, TB, and DB of female rats in the experimental group were not significantly different from those in the control group. The AST level which increased after administration in all 0.2 and 2 g/kg/day groups was significantly higher than that in the control group (*P* < 0.05), and with the increase of the dosage, there is a decreasing trend (Figures 3(a) and 3(e)). The levels of TP, ALB, and ADA in all experimental groups were higher than those in the control group, and there was a significant difference of TP and ALB levels compared with the control group (*P* < 0.05).

The results of blood glucose and blood lipid parameters of female rats showed that there was no significant difference in LDL in the administration group compared with the control group; the levels of GLU, TG, and TC were all higher than in the control group; TG levels in the 20 g/kg/day group and TC levels in the 0.2 g/kg/day group were significantly different from those in the control group; HDL levels in the 0.2 and 2 g/kg/day groups were significantly higher compared with those in the control group (*P* < 0.05); and the levels of GLU in the 0.2, 2, and 20 g/kg/day groups were significantly different from those in the control group (*P* < 0.05), as shown in Figure 3(c). The levels of BUN and UA in the female administration group were no different from the control group, but the levels of CRE were significantly higher than those in the control group (*P* < 0.05), as shown in Figure 3(d). However, the results of the blood glucose and blood lipid parameters of male rats showed that there were no significant differences between the groups, as shown in Figure 3.

### 3.5. Effect of MCM on Energy Metabolism, Oxidation, and Gastrointestinal Motility

**Figure 4:**
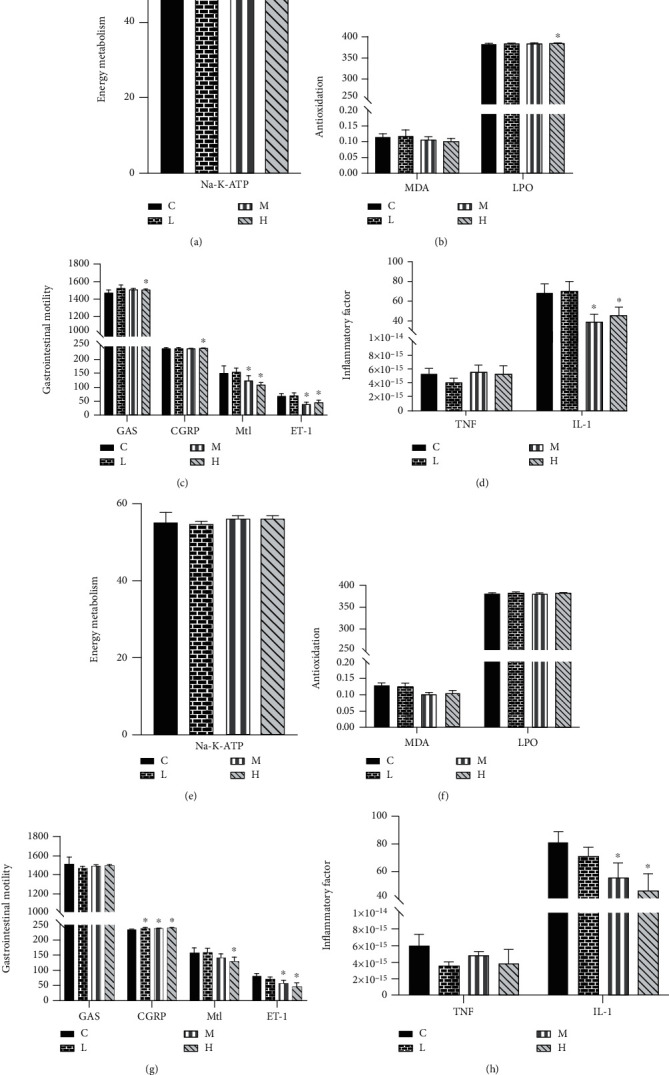
Effect of MCM on energy metabolism, oxidation, and gastrointestinal motility in rats. C: normal control group; L: water extraction of MCM 0.2 g/kg/day; M: water extraction of MCM 2 g/kg/day; H: water extraction of MCM 20 g/kg/day. ^∗^*P* < 0.05, compared with the control group; ^∗∗^*P* < 0.01, compared with the control group. MDA: malondialdehyde; LPO: lipid peroxide; GAS: gastrin; CGRP: calcitonin gene-related peptide; Mtl: motilin; ET-1: endothelin1; TNF: tumor necrosis factor; IL-1: interleukin1.

The oxidation and energy metabolism indexes of rats were measured by ELISA assay, and the results are shown in Figures 4(a)–4(d) (female rats) and Figures 4(e)–4(h) (male rats). For energy metabolism, the Na^+^-K^+^-ATP level in the serum of all experimental groups was higher than that of the control group and showed a certain dose-response relationship, but only the female experimental group showed a significant difference (*P* < 0.05). In terms of antioxidant capacity, MDA level of the administration group was not significantly different from that of the control group, but LPO level of the female 20 g/kg/day group was significantly different from that of the control group (*P* < 0.05). For gastrointestinal motility, Mtl and ET-1 levels in all 2 and 20 g/kg/day groups were significantly lower than those in the control group (*P* < 0.05), and CGRP levels in all male and female 20 g/kg/day groups were significantly different from those in the control group (*P* < 0.05). For inflammation, IL-1 levels were significantly lower in all 2 and 20 g/kg/day groups than in the control group.

### 3.6. Effects of MCM on Immunity

**Figure 5:**
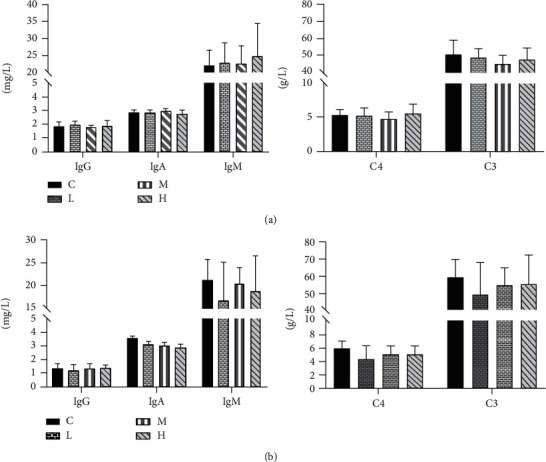
Effect of MCM on immunity in rats. C: normal control group; L: water extraction of MCM 0.2 g/kg/day; M: water extraction of MCM 2 g/kg/day; H: water extraction of MCM 20 g/kg/day. ^∗^*P* < 0.05, compared with the control group; ^∗∗^*P* < 0.01, compared with the control group. IgG: immunoglobulin G; IgA: immunoglobulin A; IgM: immunoglobulin M; C4: complement 4; C3: complement 3.

The results of the serum immunoglobulin level are shown in Figure 5. The levels of IgA, IgM, IgG, C3, and C4 did not show any difference (*P* > 0.05) compared to those in the control group.

### 3.7. Effects of MCM on Routine Urine Test

The results of routine urine tests are shown in Table 1, and there was no significant difference between the experimental groups and the control group.

### 3.8. Effects of MCM on Organ Coefficient

**Figure 6:**
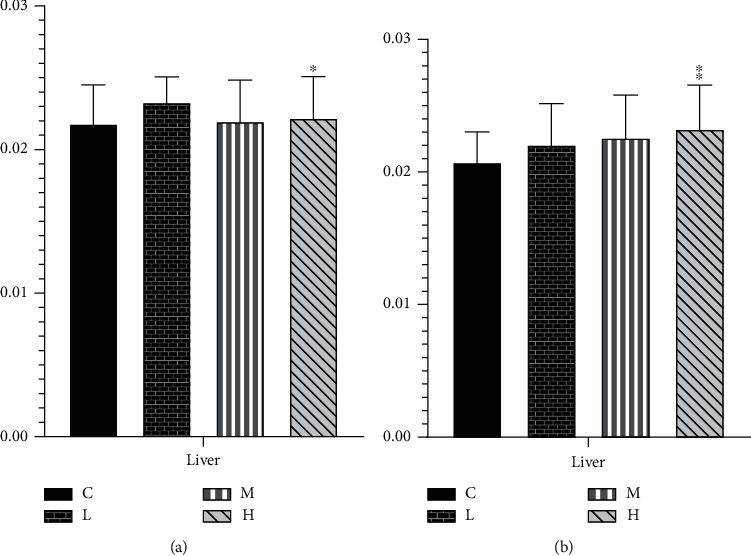
Effect of MCM on organ coefficient in rats (*n* = 10). C: normal control group; L: water extraction of MCM 0.2 g/kg/day; M: water extraction of MCM 2 g/kg/day; H: water extraction of MCM 20 g/kg/day. ^∗^*P* < 0.05, compared with the control group; ^∗∗^*P* < 0.01, compared with the control group.

As shown in Figure 6, the organ coefficients of each administration group except the liver were not significantly different from those of the control group. Compared with the normal control group, the organ coefficient of the liver of female rats is slightly lower at 2 and 20 g/kg/day (Figure 6(a)). The liver organ coefficient of male rats increases with the increase of the administered dose, which has a significant dose escalation effect, and the 20 g/kg/day group has a significant difference (*P* < 0.05) (Figure 6(b)).

### 3.9. Effects of MCM on Histopathological Analysis

After 90 days, the rats were examined by routine autopsy; no obvious changes were observed by the naked eye. Rat liver, heart, spleen, lung, kidney, stomach, intestine, testis (♂), and uterus (♀) tissues are used for routine histopathological examination. No abnormal histopathological changes were found in vital organs under an inverted microscope.

### 3.10. Effects of MCM on the Distribution of OTUs

**Figure 7:**
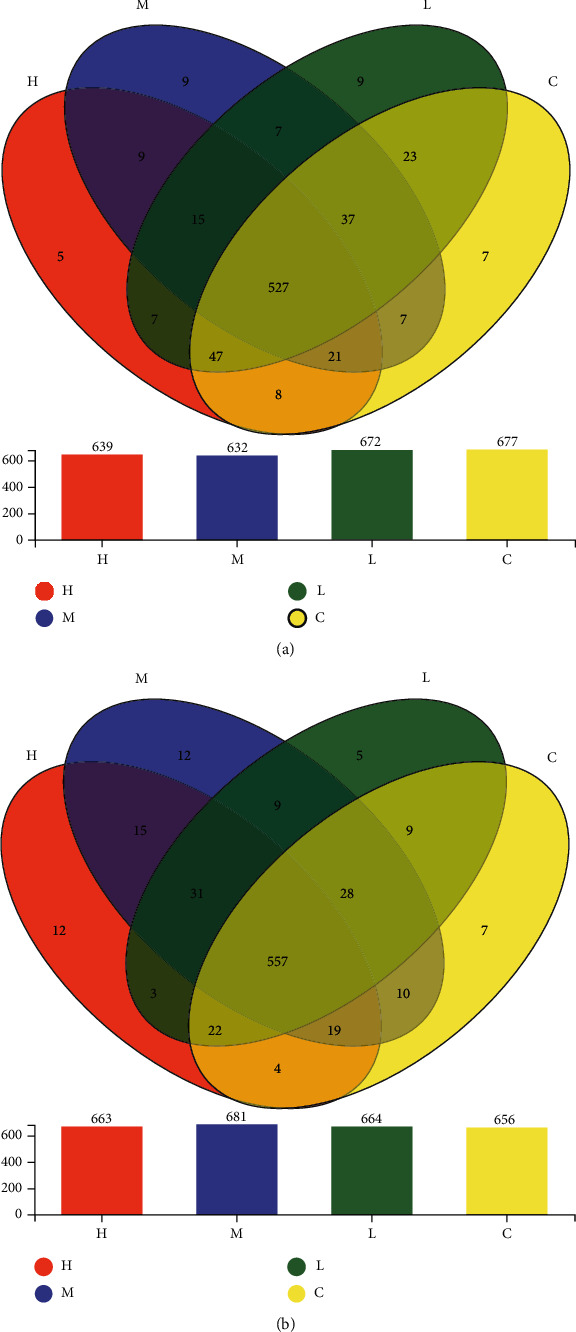
Effect of MCM on common/unique OTU number distribution in rats. C: normal control group; L: water extraction of MCM 0.2 g/kg/day; M: water extraction of MCM 2 g/kg/day; H: water extraction of MCM 20 g/kg/day.

We conducted 16S rDNA sequencing to profile gut microbiota composition and used a Venn diagram to show shared and unique OTU numbers. For female rats, there were 527 common OTUs, 7 unique OTUs in the control group, and 9, 9, and 5 unique OTUs in the 0.2, 2, and 20 g/kg/day groups (Figure 7(a)), respectively. For male rats, there were 557 common OTUs, while the unique OTUs in the control group were 7 and there were 5, 12, and 12 unique OTUs in the 0.2, 2, and 20 g/kg/day groups (Figure 7(b)), respectively.

**Figure 8:**
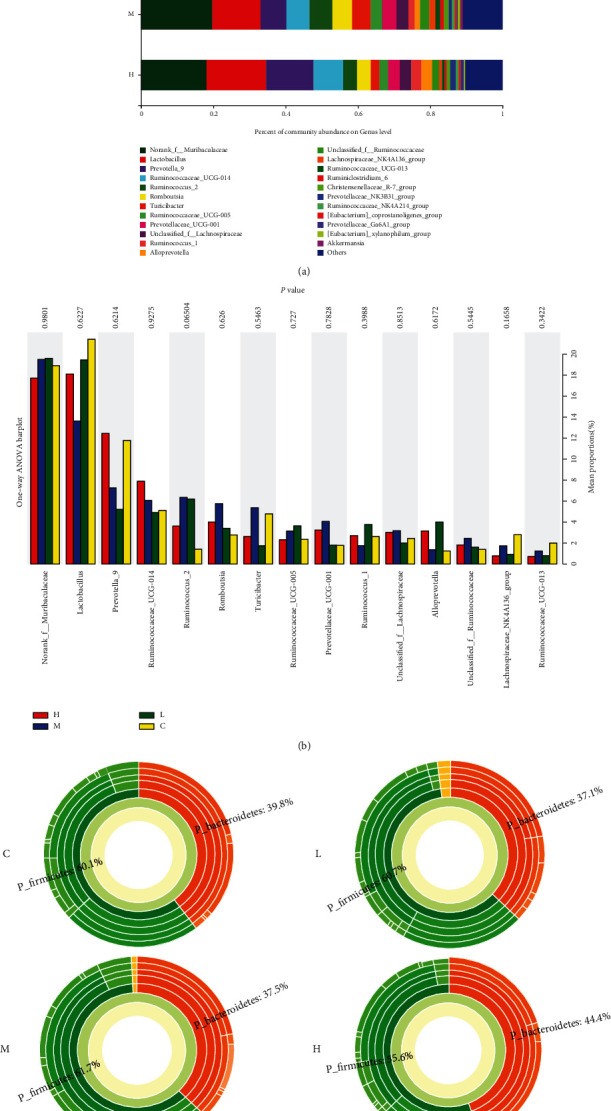
Effect of MCM on the relative abundance of gut microbiota in male rats. C: normal control group; L: water extraction of MCM 0.2 g/kg/day; M: water extraction of MCM 2 g/kg/day; H: water extraction of MCM 20 g/kg/day. ^∗^*P* < 0.05, compared with the control group; ^∗∗^*P* < 0.01, compared with the control group.

**Figure 9:**
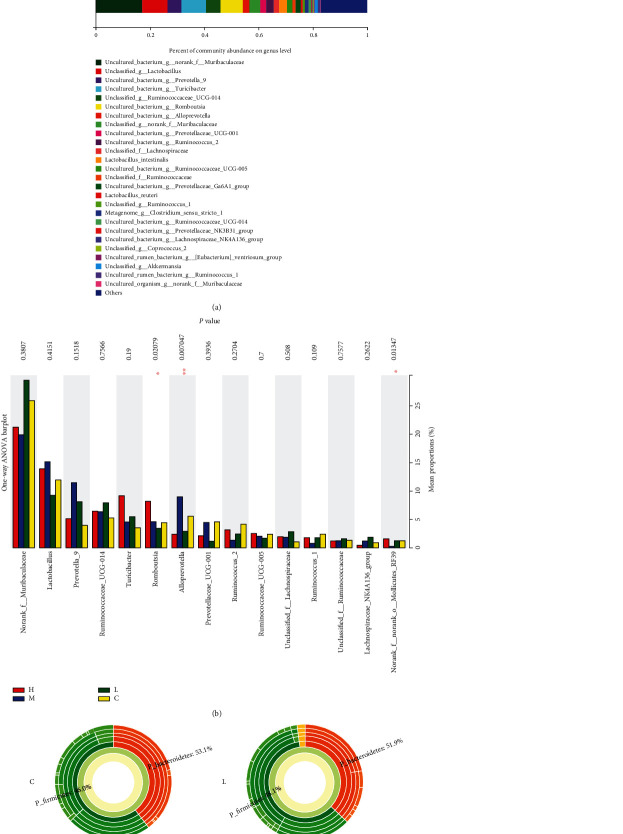
Effect of MCM on the relative abundance of gut microbiota in female rats. C: normal control group; L: water extraction of MCM 0.2 g/kg/day; M: water extraction of MCM 2 g/kg/day; H: water extraction of MCM 20 g/kg/day. ^∗^*P* < 0.05, compared with the control group; ^∗∗^*P* < 0.01, compared with the control group.

As shown in Figure 8(a), MCM 90-day feeding changes the level of intestinal flora. Females have greater influence on *Lactobacillus*, *Prevotella*, *Ruminococcus*, *Turicibacter*, *Romboutsia*, and *Alloprevotella* relative abundance. Compared with the control group, the relative abundance of *Romboutsia* and *Alloprevotella* is significantly different (*P* < 0.05), as shown in Figure 8(b). The differences between the sunburst map sets of multiple species at the bacterial genera level are shown in Figure 9(c).

The difference in intestinal microflora after 90 days of feeding of MCM is shown in Figure 9, and the results show that MCM can change the number of intestinal flora (Figure 9(a)). In the female rat groups, MCM had a greater impact on *lactobacillus*, *prevotella_9*, *ruminococeaceae_ucg_014*, *turicibacter*, *Romboutsia*, and *Alloprevotella*, but the differences between the various genera did not reach a significant difference (Figure 9(b)); the difference between the sunburst diagram groups of multilevel species at the genus level is shown in Figure 9(c).

### 3.11. Effects of MCM on Urinary Metabolism

**Figure 10:**
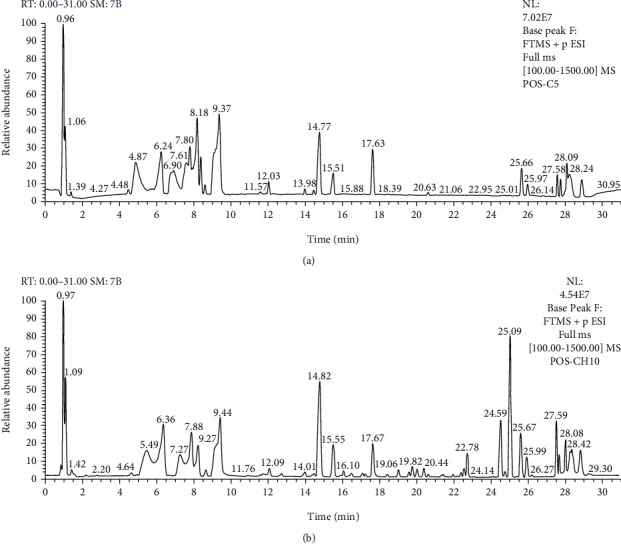
An orbitrap mass spectrometer equipped with a heated electrospray ionization (HESD) probe was coupled to the UHPLC system to intensify the chromatogram of rat urine. (a) The positive ion basic peak intensity (BPI) chromatogram of the mixed sample of the control group and the experimental group. (b) The negative ion basic peak intensity (BPI) chromatogram of the mixed sample of the control group and the experimental group.

**Figure 11:**
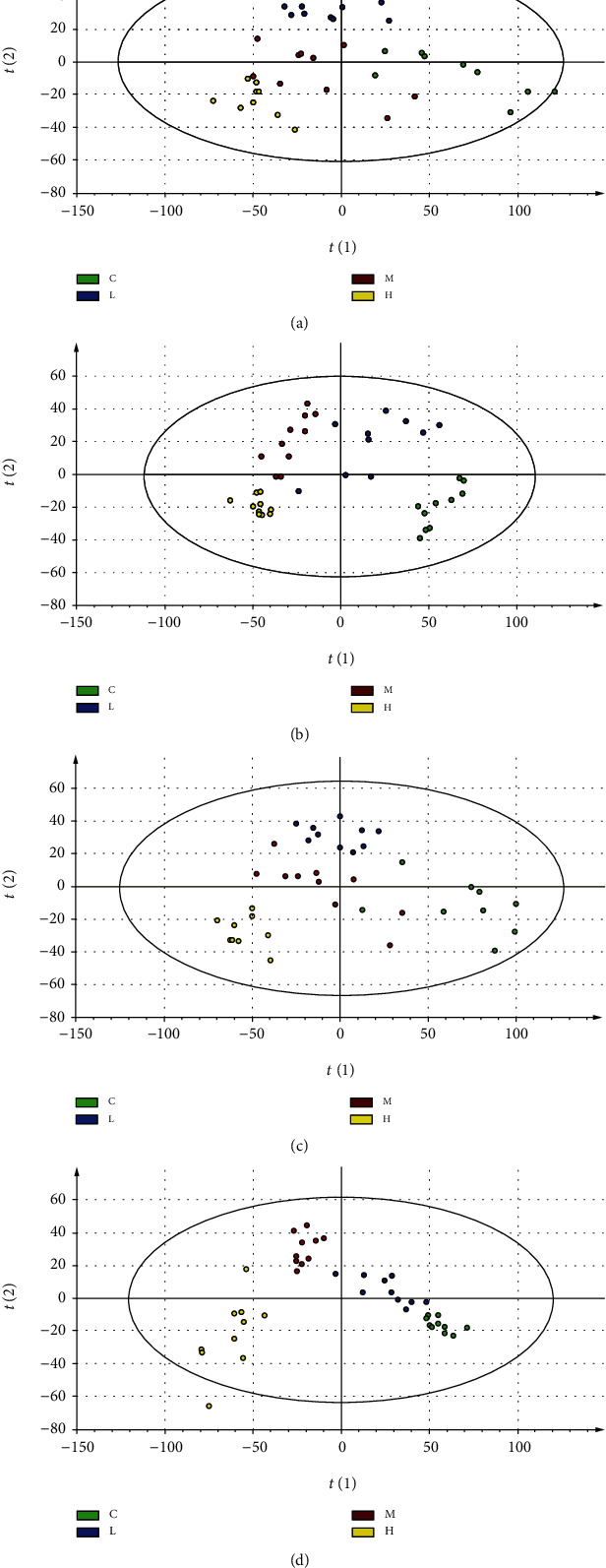
The mean PCA of the control and treated groups in positive (a, b) and in negative (c, d). (a) 12 weeks after treatment in the positive mode (R2X[1] = 0.32, R2X[2] = 0.0822); (b) 12 weeks after treatment in the positive mode (R2X[1] = 0.241, R2X[2] = 0.0919); (c) 12 weeks after treatment in the negative mode (R2X[1] = 0.31, R2X[2] = 0.091); (d) 12 weeks after treatment in the negative mode (R2X[1] = 0.273, R2X[2] = 0.0837).

The urine samples of the control and treated groups were analyzed by applying UHPLC-LTQ-Orbitrap-MS/MS in both positive and negative ionization modes to confirm the significant metabolic alterations of metabonomic profiles. The typical base peak intensity chromatogram obtained from the analysis of the positive ionization mode of the urine samples is shown in Figure 10. The PCA plots for the treated groups clearly deviate from that of the control group by the 90th day (Figure 11). The more obvious the metabolic profile changes, the greater the influence of MCM.

The potential variables were selected as the biomarkers based on variable importance in projection (VIP) value (>1) and ANOVA (*P* < 0.05). Finally, the 5 biomarkers (2 from the positive mode and 4 from the negative mode) were identified in urine (refer to Table 2) based on accurate mass, isotopic pattern, MS/MS information, and comparison with the structure message of metabolites. As shown in Table 2, indoleacetic acid, N-acetyl-L-phenylalanine, 2-hydroxycinnamic acid, kynurenic acid, and xanthurenic acid significantly decreased (*P* < 0.05). Furthermore, the urinary metabolites altered significantly in a dose-dependent manner. The 5 metabolites mentioned above demonstrated more significant changes in the 20 g/kg/day group than in the 0.2 g/kg/day group. The metabolic profiling change in the 20 g/kg/day group appeared to have more remarkable effects than that in the 2 g/kg/day group.

## 4. Discussion

The use of traditional Chinese medicine has a long history in China. Understanding the toxicity of traditional Chinese medicine is the key to rational use of traditional Chinese medicine [5–7]. Modern pharmacological studies have found that some herbal ingredients of Aristolochiaceae are toxic [8]. For example, aristolochic acid in them shows relatively strong toxicity and even irreversible permanent carcinogenicity. In recent years, more and more attention has been paid to the evaluation of the safety of traditional Chinese medicine [9–12], and there are many new methods being explored, such as network pharmacology [13], genomics [14], comparative omics, and metabonomics [15–18]. The mammalian 90-day toxicity test is an important experiment for safety evaluation, and the traditional 90-day feeding test has relatively few end points and narrow coverage. Nowadays, some toxicologists have begun to explore the use of invertebrates as an alternative to mammals for long-term studies [19]. We added intestinal microbial diversity detection and urine mass spectrometry to the traditional 90-day feeding test analysis in order to obtain more comprehensive data for safety evaluation of subjects.

The weight of experimental animals during their growth and development is one of the most basic and sensitive indicators to comprehensively reflect the health status of animals [20], so it is an important observation indicator in the toxicity test, as well as the balance of lipid metabolism. In the 90-day subchronic toxicity study of Elsholtzia Maxim, it was shown that it had no significant influence on the weight and growth of rats. However, the weight of male rats from the second week showed that the weight of the administration group was slightly lower than that of the normal control group, and the TC and LDL levels of the male administration group were significantly lower than those of the normal control group, and the HDL level increased. The results are in line with previous studies that MCM has the effect of lowering blood lipids [21].

The blood routine index is an important index to reflect whether the test substance has affected the hematopoietic function. The results of this experiment showed that the number of red blood cells in female rats with 0.2 and 20 g/kg/day and male rats with 2 g/kg/day increased after 90 days, but no obvious dose-effect relationship was observed, so comprehensive analysis showed that the feeding experiment of MCM for 90 days had no obvious influence on the blood routine of rats.

In order to improve the overall understanding of the safety of traditional Chinese medicine, we increased the detection of serum energy metabolism, oxidation index, and gastrointestinal motility index. Motilin (Mtl) is one of the hormones of the digestive system; its function is to increase the migrating motor complex (MMC), promote gastrointestinal movement, and stimulate the secretion of pepsin, while promoting gastric emptying [22, 23]. The results of this experiment showed that Mtl levels in all the treatment groups decreased, and the Mtl levels in the 2 and 20 g/kg/day groups were significantly lower than those in the control group. Calcitonin gene-related peptide (CGRP) is a neuropeptide that is distributed in the brain and gastrointestinal system and has a variety of biological effects; it can protect gastrointestinal function by inhibiting gastric acid secretion, increasing gastric mucosal blood flow, slowing gastrointestinal movement, inhibiting inflammatory reaction, antagonizing free radical damage, and regulating gastrointestinal hormone secretion. In this experiment, it was found that the CGRP level of male rats in the 0.2, 2, and 20 g/kg/day groups and female rats in the 20 g/kg/day group increased, and there were significant differences with the control group. It was reported that the increase of the CGRP level could reduce the release of inflammatory factors TNF and IL-1 [24]. Therefore, the results show that MCM can inhibit the release of inflammatory cytokines and play an important role in regulating local inflammation. The results are in line with the clinical treatment of acute gastroenteritis and abdominal pain vomiting and diarrhea by MCM, which may have good resistance to intestinal accumulation heat and enhance the characteristics of intestinal peristalsis.

The levels of ALT, AST, ADA, and CHE can reflect the damage and severity of liver cells. The biochemical indicators of liver function such as TP, ALB, and CHE can reflect the anabolic function of cell protein. This indicates a decrease in ALT level in serum liver injury [25, 26]. The levels of TB, DB, and associates can reflect the function of liver excretion and secretion and detoxification; ALP and GGT levels can reflect cholestasis. AST and ALT are very important enzymes in the metabolic process of the body. When liver cells are damaged, AST and ALT are released, increasing the levels of AST and ALT in the serum. However, when the increase of these two indicators does not exceed twice, there is no toxicological significance [27]. This study found that AST and ALT increased in both the female and male administration groups, and the liver organ coefficient was larger than that of the normal control group. It shows that MCM has an effect on the liver, but it is not toxic, and the pathological section shows normal.

BUN, CRE, and UA were the indicators of renal function. BUN is the end product of amino acid and protein metabolism of the body and is the main component of nonprotein in the blood. When the kidney is injured, the excretion of BUN decreases, leading to an increase in the level of BUN in the serum. CRE is a kind of small molecule product produced by muscle metabolism in the body, which can be filtered and excreted by the kidney. CRE level is an important indicator to detect the detoxification ability of the kidney [28]. In this experiment, it was found that the effects of 90 days of feeding of water extracted from MCM on renal function had no significant toxicological significance.

Being rich in the amount and type of gut microbes not only helps digestion and absorption of nutrients, it also has a huge impact on the ADME process of exogenous chemicals. At the same time, these metabolites can adversely affect the abundance and diversity of the gut microbes, and analyzing the distribution of intestinal flora is the innovation of this experiment. From the results (Figure 7), the distribution of intestinal flora in the administration group and the control group has changed. Compared with the normal control group, the flora with a significantly higher relative abundance of the MCM administration groups are *Prevotella*, *Ruminococcaceae*, and *Romboutsia* (Figures 8 and 9). In terms of urine metabolomics, it was found that the level of uric acid in the urinary metabolites of the administration group was significantly different from that of the normal control group; the results are similar to previous studies. Previous studies have found that intestinal flora is involved in the regulation of uric acid excretion. Short-chain fatty acids (such as butyrate) regulate the proliferation and repair of intestinal epithelial cells, change the number and distribution of uric acid transporters in the body, and affect the transport and excretion of uric acid. Lim et al. [29] conducted a horizontal study and analysis of fecal microbes in the general population of Korea and a two-year longitudinal study and analysis of fecal microbes in identical twin pairs and confirmed that the level of human blood uric acid and the dominant type of intestinal flora are closely related. Among them, the dominant intestinal type of *Prevotella* (the main decomposition products are butyric acid, succinic acid, lactic acid, etc.) has lower blood uric acid than the dominant intestinal type of Bacteroides. In 2016, Stern et al. [30] used broad-spectrum gene sequencing technology to compare and analyze the stools of 23 patients with kidney stones and 6 in the general population and also found the correlation between hemorrhagic uric acid and the abundance of Prevotella and Bacteroides. In the same year, Guo et al. [31] studied the intestinal flora and metabolites and found that the type and quantity of intestinal flora in patients with gout and the decrease in the content of broken chain fatty acid butyric acid are associated with xanthine dehydrogenase and uric acid in the intestine. The decrease in the level of uric acid in the urine may be caused by changes in the distribution of intestinal flora.

N-Acetyl-L-phenylalanine is the product of phenylalanine N-acetyltransferase in the phenylalanine metabolic pathway. Changes in the level of N-acetyl-L-phenylalanine indicate that exposure to MCM will affect the metabolism of phenylalanine. Indole acetic acid is the starting point and central compound of the tryptophan metabolism pathway. Tryptophan metabolism is closely related to the central nervous system and immune regulation and plays an important role in maintaining the normal physiological functions of the central nervous system. Therefore, the change of indole acetic acid content suggests that MCM can interfere with the tryptophan metabolism of normal rats. Previous studies have shown that tryptophan and its metabolites are involved in the development of liver steatosis and steatohepatitis [32]. Changes in ALT and AST levels may be related to the metabolic pathways of tryptophan.

## 5. Conclusion

Combining the above detection indicators, it is shown that the tested drug Mosla chinensis Maxim water extract is actually nontoxic and safe for clinical application. The results provide a scientific basis for the further application of MCM.

## Figures and Tables

**Table 1:** Effect of MCM on urine routine in rats.

	♂				♀			
Index	C	L	M	H	C	L	M	H
Occult blood	2/10	2/10	0/10	0/10	2/10	2/10	1/10	1/10
Protein	1/10	1/10	0/10	0/10	1/10	1/10	0/10	1/10
Bilirubin	2/10	1/10	0/10	1/10	1/10	1/10	1/10	1/10
Urobilinogen	<16	<16	<16	<16	<16	<16	<16	<16
Ketone body	0/10	0/10	0/10	0/10	0/10	0/10	0/10	0/10
Leukocyte	0/10	0/10	0/10	0/10	0/10	0/10	0/10	0/10

C: normal control group; L: water extraction of MCM 0.2 g/kg/day; M: water extraction of MCM 2 g/kg/day; H: water extraction of MCM 20 g/kg/day.

**Table 2:** Effects of MCM on toxic biomarkers in rats.

Retention time (min)	Measured mass (Da)	Calculate mass (Da)	Error (ppm)	Scan mode	Identity	Elemental composition	*P* value	Fold change
12.67	176.0706	176.0706	0	+	Indoleacetic acid	C10H9NO2	5.8 × 10^−5^	2.769
10.26	208.0970	208.0968	0	+	N-Acetyl-L-phenylalanine	C11H13NO3	1.9 × 10^−4^	3.252
7.55	162.0571	163.0516	6	−	2-Hydroxycinnamic acid	C9H8O3	3.1 × 10^−9^	4.282
5.21	188.0362	188.0353	4	−	Kynurenic acid	C10H7NO3	1.2 × 10^−9^	4.699
3.94	204.0312	204.0302	4	−	Xanthurenic acid	C10H7NO4	2.2 × 10^−8^	3.355

*P* value: group of 20 g/kg/day compared with the control group at the 90^th^ day. Fold change was calculated by dividing the mean of the peak intensity of each metabolite from the 20 g/kg/day group relative to the control group at the 90^th^ day.

## Data Availability

The data used to support the findings of this study are available from the corresponding author upon request.

## Abbreviations

MCM: Mosla chinensis Maxim
RBC: Red blood cell
WBC: White blood cell
HGB: Hemoglobin
PLT: Platelet
ALB: Albumin
TBA: Total bile acid
ALT: Alanine transaminase
TP: Total protein
ALP: Alkaline phosphatase
ADA: Adenosine deaminase
AST: Aspartate aminotransferase
CHE: Cholinesterase
TB: Total bilirubin
DB: Direct bilirubin
GGT: Glutamyl transferase
LDL: Low-density lipoprotein
TG: Triglyceride
TC: Total cholesterol
HDL: High-density lipoprotein
GLU: Glucose
BUN: Blood urea nitrogen
CRE: Creatinine
UA: Uric acid
MDA: Malondialdehyde
LPO: Lipid peroxide
GAS: Gastrin
CGRP: Calcitonin gene-related peptide
Mtl: Motilin
ET-1: Endothelin1
TNF: Tumor necrosis factor
IL-1: Interleukin1
IgG: Immunoglobulin G
IgA: Immunoglobulin A
IgM: Immunoglobulin M
C4: Complement 4
C3: Complement 3
TCM: Traditional Chinese medicine.

## Supplementary Materials

Supplementary Materials Parameter of elution gradient in positive and negative ionization mode. Solvent A: 0.1% aqueous formic acid; solvent B: acetonitrile.
