# Supplementary material for: The Subchronic Toxic Effects of Mosla chinensis Maxim in Normal Rats
Source: Biomed Res Int. 2020 Dec 28;2020:4521586. doi: 10.1155/2020/4521586 (PMC7814957; doi:10.1155/2020/4521586)
Supplement: Supplementary Materials — Parameter of elution gradient in positive and negative ionization mode. Solvent A: 0.1% aqueous formic acid; solvent B: acetonitrile. [file 4521586.f1.docx]

**Table S1**

**Parameter of elution gradient in positive and negative ionization mode**

| Positive/negative ionization mode | | | |
| --- | --- | --- | --- |
| Time(min) | Flow Rate(ml/min) | A% | B% |
| Initial | 0.300 | 95.0 | 5.0 |
| 2.000 | 0.300 | 95.0 | 5.0 |
| 25.000 | 0.300 | 55.0 | 45.0 |
| 27.000 | 0.300 | 0.0 | 100.0 |
| 27.000 | 0.300 | 95.0 | 5.0 |
| 31.000 | 0.300 | 95.0 | 5.0 |

Solvent A: 0.1% aqueous formic acid; Solvent B: acetonitrile
